# Developing national strategies for reaching men with HIV testing services in Tanzania: results from the male catch-up plan

**DOI:** 10.1186/s12913-019-4120-3

**Published:** 2019-05-20

**Authors:** Donaldson F. Conserve, Jumanne Issango, Andrew M. Kilale, Bernard Njau, Patrick Nhigula, Peter Memiah, Gaspar Mbita, Augustine T. Choko, Akeen Hamilton, Gary King

**Affiliations:** 1 Department of Health Promotion, Education, and Behavior, Arnold School of Public Health, University of South Carolina, Columbia, SC USA; 2 Tanzania Commission for AIDS (TACAIDS), Dar es Salaam, Tanzania; 3 National Institute of Medical Research, Dar es Salaam, Tanzania; 4 Kilimanjaro Christian Medical Centre, Moshi, Tanzania; 5 Darla Moore School of Business, University of South Carolina, Columbia, USA; 6 University of West Florida, Pensacola, FL USA; 7 Jhpiego Tanzania, Dar es Salaam, Tanzania; 8 Malawi-Liverpool Wellcome Trust Clinical Research Programme, Blantyre, Malawi; 9 Department of Biobehavioral Health, Pennsylvania State University, University Park, State College, PA USA

**Keywords:** Men: HIV testing, Barriers, Intervention, National, Strategies, Tanzania

## Abstract

**Background:**

According to the 2016–2017 Tanzania HIV Impact Survey, 55% of men diagnosed with HIV during the survey self-reported that they were unaware of their HIV status. As a response, the Government of Tanzania launched a Test and Treat campaign in June 2018 with a focus on reaching men and developed the 2018-2020 Male Catch-Up plan. This article reports (1) the enablers and barriers of HIV testing services (HTS) uptake among men (2) and describes the strategies that were proposed as part of the Male Catch-Up Plan to address some of these barriers.

**Method:**

Qualitative in-depth interviews were conducted with 23 men in Dar es Salaam to explore HTS enablers and barriers. To develop the Male Catch-Up Plan strategies, a desk review of published studies, and analyses of national implementers of HIV/AIDS interventions were conducted. An additional 123 interviews were also carried out with key implementers of HIV/AIDS interventions, healthcare workers, secondary school boys and members of the community in Iringa and Tanga.

**Results:**

Enablers of HTS included the desire to check one’s health, high HIV risk perception, wanting to protect oneself if tested negative, and being encouraged by their sexual partners. Barriers of HTS were fear of a positive test result, and low HIV risk perception. Proposed strategies from the Male Catch-Up Plan to address these barriers included non-biomedical and biomedical approaches. Non-biomedical strategies are social and cultural approaches to promote an enabling environment to encourage health seeking behavior, safe behavior, and providing peer education programs and social marketing to promote condoms. Biomedical approaches consisted of expanding targeted HIV testing, HIV self-testing, and integrating HIV services with other health services.

**Conclusion:**

A number of barriers contribute to the low uptake of HTS among men in Tanzania. National strategies have been developed to address these HTS barriers and guide the national Test and Treat campaign focusing on increasing HTS uptake among men.

**Electronic supplementary material:**

The online version of this article (10.1186/s12913-019-4120-3) contains supplementary material, which is available to authorized users.

## Background

According to the 2017 Joint United Nation Program on HIV/AIDS (UNAIDS) report, sub-Saharan Africa (SSA) accounted for 64% of new HIV infections [1]. In Tanzania, existing evidence indicates that HIV incidence and prevalence have declined and stabilized [2, 3]. The incidence of HIV infection in the age group 15–49 years peaked at 1.34% in 1992, declined rapidly down to 0.64% in 2000, and steadily declined further to 0.29% in 2015 [2]. HIV prevalence has also declined in Tanzania from 7.0% in 2003/04 to 5.5% 2016 [2, 3]. However, there exists age and gender differentials in HIV prevalence in Tanzania, with the prevalence among young women aged 25–29 being three times higher compared to young men in the same age group [2, 3]. In addition, there is a marked rural-urban and sex differences in HIV prevalence, with higher prevalence existing among both men and women in urban (7.2%) than rural areas (4.3%) [2, 3]. In order to continue the decline of HIV incidence and lower the prevalence among young women, more efforts are needed to reach the male partners of young women in Tanzania with HIV prevention programs.

HIV testing services (HTS) are an essential component of HIV/AIDS control programs globally and an entry point of the HIV care and treatment cascade [4]. Benefits associated with HTS include, but are not limited to, early detection and initiation of HIV care and treatment [4]. Efforts to provide quality care to people living with HIV (PLWH) in Tanzania has increased since the Government of Tanzania started implementing the HIV/AIDS Care and Treatment Plan in 2003 [5]. HIV prevention, care, treatment, and support services are provided by the National AIDS Control Program (NACP) through the Ministry of Health, Community Development, Gender, Elderly, and Children (MOHCDGEC) and the Tanzania Commission for AIDS (TACAIDS) manages the multi-sectoral HIV/AIDS response [5]. In particular, HTS are provided for free at both the health facilities and community settings while HIV Care and Treatment Centers (CTCs) are established in hospitals as well as in health centers and dispensaries [6]. In spite of the free antiretroviral treatment (ART) and the scale up of HTS, care and treatment in healthcare facilities and communities across the country, 55% of men living with HIV (MLWH) self-reported that they were unaware of their HIV status during the Tanzania HIV Impact Survey conducted in 2016–2017 [7].

General barriers contributing to the limited uptake of HTS include HIV-related stigma and discrimination [8–10] lack of confidentiality, fear of an HIV-positive test result, [11] and increased waiting time to obtain a test result [12]. Contextual barriers associated with low uptake of HIV testing includes: a lack of resources (e.g., HIV testing reagents), long-waiting time and long queues at HIV testing points, lack of counselors, and inconvenient hours of operation [13–15]. Among men in particular, their reluctance to test for HIV is influenced by a masculinity ethos, which prevents them from expressing emotions in public and create the notion that HIV testing is a woman’s domain [16–20]. Men are also reluctant to test due to the heightened sense of risk related to extramarital relationships and resultant fear of receiving a positive diagnosis [11, 16, 20, 21]. These barriers impede countries, such as Tanzania, from reaching the UNAIDS 90–90-90 targets among men, which called for 90% of people living with HIV (PLWH) to know their status, 90% of PLWH to receive antiretroviral therapy (ART), and 90% of those on ART to achieve viral suppression by the year 2020 [22].

In an effort to make progress towards the UNAIDS 90–90-90 targets, on December 1st 2017 during the World AIDS Commemoration Day in Dar es Salaam the Prime Minister of Tanzania requested TACAIDS to develop a targeted male engagement plan to increase HIV testing and enrolment among men [23]. As a response, TACAIDS and other partners, including but not limited to, NACP, UNAIDS Tanzania, Benjamin Mkapa Foundation (BMF), National Institute for Medical Research (NIMR) at Muhimbili Medical Research Centre, developed strategies for engaging men in HTS as part of the 2018–2020 Male Catch-Up Plan [23]. Building on this momentum, the Prime Minister collaborated with national and international partners to launch a national Test and Treat campaign in June 2018 called *Furaha Yangu! (My Happiness!)*, with a focus on reaching men and boys to test for HIV and learn their HIV status as part of the Fast-Track Framework [23]. The Fast-Track Approach framework or Approach of ending AIDS by 2030 calls for specific targets at both global and country level targets and aims to ensure that people at risk test for HIV and initiate treatment if they are positive, which can in turn lead to viral load suppression [24]. The focus of the campaign to reach men and adolescent boys with HTS is aligned with the Fast-Track Approach and is being informed by the strategies TACAIDS and collaborators developed to engage men that are included in the 2018–2020 Male Catch-Up Plan [23].

The evidence from our research with men in Dar es Salaam over the past decade also supported the need to develop strategies to reach men with HTS in Tanzania [25, 26]. Based on the low HIV testing rate reported by men in our previous research [26], our team had conducted qualitative formative research to assess enablers and barriers of HTS uptake among men as part of a larger study funded by the National Institute of Health (NIH) [27]. Therefore, in this paper, we utilize data from our qualitative research with men and the Male Cath-Up Plan to report the: 1) enablers and barriers of HTS among the men; and 2) national strategies developed to address HTS uptake barriers.

## Methods

### Data sources

The data analyzed in the current paper are from the formative research conducted as part of the NIH-funded project in Dar es Salaam and the 2018–2020 Male Catch-Up Plan. The development process for the Catch-Up Plan included: 1) a desk review of published studies, reports, and analysis of data from national level implementers of HIV/AIDS interventions, and stakeholders; 2) interviews conducted with healthcare workers, key implementers of HIV/AIDS interventions, and members of the community, including leaders and in-school adolescent boys, in Iringa rural and Tanga Municipal Councils and; 3) meeting with stakeholders to review and contribute to the plan.

#### Formative research Data collection

The protocol for the RCT and a description of the data collection process for the formative research are published elsewhere [27, 28]. Briefly, the RCT was conducted within 60 camps, with 30 camps assigned to the treatment group and 30 to the control group [27]. A total of 1249 men completed the baseline behavioral assessment in fall 2013 and 978 of these men completed the 12-month follow-up in 2015 for the larger RCT [29, 30]. A total of 23 participants from the RCT were contacted and interviewed between November–December 2015 for the formative research [28]. The interview guide was piloted and modified during meetings held by the study team to discuss the feedback received during the piloting phase. Sample interview guide questions included: *Can you tell me about the reasons you decided to get tested for HIV? Can you please tell me about some of the reason(s) you have not tested for HIV? What goes through your mind when you think about getting tested for HIV?* Further details about the interviews and participants’ compensation can be found elsewhere [28].

#### Male catch-up plan development process

Google Scholar and PubMed were used to search for published studies during the process of developing the Catch-Up Plan [23]. In addition, technical reports, and published program reports were provided by key implementers of HIV/AIDS program in Tanzania to facilitate the review. The stakeholders included but are not limited to community health workers, community leaders, and representatives from the NACP, World Health Organization (WHO), Center for Diseases Control and Prevention (CDC), United Nations Population Fund (UNFPA), ICAP, National Council for People Living with HIV and AIDS (NACOPHA) [23]. A total of 124 interviews were conducted between March and May 2018 by two interviewers [23]. After the interviews, notes were compared and consensus were reached for the final report. The objective of the interviews was to assess bottlenecks towards engagement of adult men and young boys in the utilization of HIV/AIDS services and develop a Catch-Up Plan to scale up their engagement and linkage to care and support [23]. The implementers were therefore interviewed to gather their experience and opinion on each of the specific objectives. Their selection was based on their previous involvement in the implementation and or evaluation of the effectiveness of strategies targeting to improve male engagement in HIV/AIDS services including HTC, care and treatment. The main question from theses interviews that we focus on in this paper is the following: *How do you think the barriers/factors affecting uptake of HIV counseling and testing among adolescent boys and adult males has to be intervened (see* Additional file 1*)?* Once the interviews were complete, the Plan was prepared, reviewed, and presented three times during a series of stakeholders meetings organized by TACAIDS to gain further consensus on the plan.

### Ethical approval

The study was approved by the University of North Carolina at Chapel Hill Institutional Review Board and the Muhimbili University of Health and Allied Sciences. Written informed consent was obtained from all participants prior to data collection.

### Data analysis

We used a priori codes: enablers and barriers of HIV testing for the analysis**.** Rigor and trustworthiness of the study findings were established by having the interviewers also transcribe the interviews to ensure the transcripts were transcribed accurately. We also held meetings with the interviewers to discuss the findings and confirm the notes taken during the interviews were consistent with the transcripts and findings. More information about the data analysis is available elsewhere [28]. Briefly, a formal codebook consisting of deductive codes was created and used to apply deductive codes and identify emerging codes using the ATLAS.ti 7.5 qualitative analysis software [28]. The research team met throughout the data analysis period to reach consensus on code definitions, code application, and selecting quotations for illustrative purposes [28]. We reached data saturation when no additional codes were discerned.

## Results

The mean age was 27.3 years (± 6.5), ranging from 20 to 51 years old (Table 1). More than half (52%) of the men had a primary education. Nearly half of the men were married (*n* = 11, 48%), married or cohabiting (*n* = 11, 48%) and the majority (65%) of them were self-employed. Nearly half (*n* = 10, 48%) of the participants had not tested for HIV test in the past 12 months. Enablers of HTS included the desire to *check one’s health*, *high HIV risk perception, wanting to protect oneself if tested negative*, and *being encouraged by their sexual partners*. Barriers of HTS uptake were *fear of a positive test result* and *low HIV risk perception*.

**Table 1:** Participant demographic characteristics

	Mean (SD) (Min -Max)	Frequency	%
Age	27.3 (+ 6.5) (20–51)		
Education			
No formal education		1	4
Primary		12	52
Secondary		9	39
Higher than secondary		1	4
Marital status			
Single		11	48
Married/cohabiting		11	48
Girlfriend		1	4
Employment			
Employed		2	9
Self-employed		15	65
Unemployed student		1	4
Unemployed non-student		5	22
HIV test in the past 12 months			
No		10	48
Yes		12	52

### HIV testing enablers

#### To check One’s health

The motivations for testing varied for personal, social, and professional reasons, with the most common reason being to *check their health* as stated in the following quote:

One of the reasons which made me to test for HIV was to know my health if I am HIV-positive or not. (Participant #34013)

#### High HIV risk perception & Partner’s infidelity

Aside from *checking one’s health* because of having a habit of testing another enabler was the men’s perception of their high risk of acquiring HIV due to previous risky sexual encounters. In addition, to having multiple sexual partners themselves, some of the participants mentioned that their female partners may also have other partners even if they are married. The risky sexual behaviors men engaged in encouraged them to *check their health* by testing for HIV. In this case, the desire to check one’s health may increase overtime based on the frequency of risky sexual behaviors and eventually leads one to move from desiring to check his health to taking the step to test. Participant #34013 described how his experience with unsafe sex and multiple sexual partners over a period prompted him to test for HIV in order to know his health and confirm whether he had acquired HIV or not:

Before that as you know when you live a bachelor life you find that today you make love with this one and tomorrow with that one… You find that even if you had planned to use protection but at the end of the day you find protection has no meaning…You just find yourself feeling that I have passed on those ways for a long time and perhaps other ways were not safe. (Participant #34013)

Aside from testing for HIV because of having multiple sexual partners, they also reported that their female partners may have other male partners as well which may increase their risk, especially if they are having unprotected sex with those partners. One participant described how the likelihood that he and his partner may have multiple sexual partners placed him at risk and thus motivated him to check his health and protect himself:

I test for HIV frequently in order to know my health status entirely… You may find I have one partner and she does not know that I have another woman, so those women also may have other partners apart from me so you end up with a huge network of partners, in such a way it makes you want to have sex while protecting yourself because you do not know. (Participant #1001)

#### Desire to protect oneself

For some men with high HIV risk perception, testing for HIV and learning that they are HIV-negative motivated them to reduce their risky sexual behavior. They seek HIV testing in order to confirm if they should protect themselves moving forward, with some of them reporting that they reduced their sexual partners and/or engaged in protected sex because they learned of their negative HIV status and wanted to protect themselves. One participant described how he carried condoms with him on the road in order to protect himself since he knows his status. He also provided an example of how knowing his HIV-negative status prompted him to use a condom recently:

You know, as a driver one gets to travel a lot during which you meet a lot of different people… For instance few days ago on my way back from Songea, I met two girls…I liked one of them so I dealt with her… but I used condoms because I know my status. (Participant #12029)

As described above, high HIV risk perception resulting from engaging in unsafe sex and having multiple sexual partners sometimes motivated men to learn their HIV status and protect themselves in order to lower their chances of contracting HIV.

#### Sexual partner influence

For other participants with concerns about how their previous sexual behaviors may have put them at risk, the decision to check their health was more difficult and required encouragement from their sexual partners. One participant who eventually tested for the first time during the intervention after receiving HIV prevention training in the camp and encouragement from his wife, described his challenges and observations prior to testing. His prior risky sexual behavior combined with the stigma his friend experienced made it difficult for him to test. He eventually tested after his wife tested first as illustrated in the following quote:

I had to tell my wife to go first…When she came back and I asked her she only told me that I also needed to go but I figured she was ok so the second time which is when she delivered the child we went together and it was all ok. (Participant #30011)

Similar to the previous participant, a number of men were encouraged to test by their partners and went to test with them.

One participant mentioned the following about his reason for testing:

I was not alone but I was with my partner who we love each other and we discussed about going to check our health because we have been together for about more than one year, therefore, we said let us go to check our health and we went to check and found we are fresh. (Participant #1003)

The encouragement to test from a partner may not always lead to testing together but some of the men eventually tested alone as described in the following quote:

There was a time when my wife was pregnant and she told me go to test together but I didn’t go, so when I decided to go to test I went alone. (Participant #34015)

### HIV testing barriers

#### Fear of testing positive

Among the men who had never tested, the main deterrent of testing was the fear of a positive test result and the ensuing worry as described in the following quote by one man:

I mean that fear of waiting for the results, don’t you know that when you go there you may be told that you are HIV-positive or negative, so I was not prepared… It is that fear that what will happen if I am found to be HIV positive. (Participant #1013)

Another participant mentioned his lack of preparation to deal with a potential HIV-positive test result:

Everything requires preparations so I have not prepared myself to test… I am not yet prepared and my soul is not yet ready… You know every human being likes to know their health status, if it is malaria you simply got to check but HIV and how big it is makes you think about what will happen when you will go to test and the results turn out to be bad and you end up saying I am infected. (Participant #22004)

#### High HIV risk perception

While some participants’ risky sexual behaviors motivated them to test for HIV in order to check their health in the previous section, others who have not tested mentioned that their risky sexual behavior created a fear that prevented them from testing. Participant #22004 who reported his fear of testing positive provided an example of the risky behaviors that prevented him and some of his peers from testing:

I mean when you drink alcohol and you get aroused then you look for a woman and fornicates and thereafter you have finished the act you start blaming yourself why did you do such a thing… If you have slept with three women in a week, it is hard to know. Although of the three, you only used a condom with one only. You see, so it is obvious you will become worried about it, that what if I test and... So when it comes to go for a test, it becomes a battle you end up not going. (Participant #22004)

### Low HIV risk perception

One other less common reason that deterred men from testing included feeling healthy and the absence of HIV symptoms:

Since I have not seen any problem with my body that is related to HIV, that is why I have not tested but If I did see any symptoms I would have gone...When I think of going to test for HIV, I do not think of anything because I do not have any fear. (Participant #10008)

## Discussion

This study assessed the enablers and barriers of HIV testing for men in Tanzania. The findings revealed that HIV testing enablers included the need to check one’s health, high HIV risk perception, the desire to protect oneself from contracting HIV if found to be negative, and being influenced by their partner. Similar HIV testing enablers have been found for men in other Sub-Saharan countries [20, 31, 32]. While more than half of the men had been tested in this study, those who had not been tested mentioned that fear of testing positive, both high and low perceived HIV risk, and lack of symptoms were barriers to HIV testing. These findings parallel the reasons men reported for not testing for HIV in previous studies [20, 31, 32] and in Iringa and Tanga, the two regions where healthcare workers, community representatives, and in-school boys were interviewed for the Male Catch-Up Plan [23].

The need to check one’s health and desire to protect oneself if tested negative as enablers of HIV testing is consistent with findings from the NIH-funded Healthy Beginning Initiative (HBI) study that was designed to address barriers of HIV testing among women and their male partners in Nigeria [33]. Findings from the HBI study revealed that men and their partners perceived HIV testing as an opportunity to know one’s HIV status and, if negative, reduce risky sexual behaviors to prevent HIV acquisition [34]. Taking step to learn one’s HIV status, especially for men with a history of risky sexual behavior, can sometimes be delayed for different reasons, including but not limited to, the potential threat to their masculinity, HIV-related stigma, and the belief that he might already be infected and does not need to protect himself [32, 35, 36]. Thus, the fact that some men in our study who perceived themselves to be at high risk wanted to learn their HIV status highlights a promising change in behavior that may help to encourage men who test negative to protect themselves and those who test positive to initiate treatment and potentially prevent HIV acquisition or transmission.

Our finding that sexual partners played a role on men’s HIV testing uptake in this study also corroborates another recent study describing how men’s sexual partners in Uganda influence their HIV testing behavior [37]. Consistent with our study, men in Uganda reported that the distrust they had for their sexual partners led them to test for HIV, especially when the men had been away for a long period of time [37]. In other cases, men who had been away for a long time or suspected to have multiple sexual partners shared that they tested because their women did not trust them and requested them to test [37]. The positive response from men after being requested to test by their sexual partners contrasts with an older study in Uganda that found that some men responded negatively when their wives suggested HIV testing [35]. In addition, it was not common for women to discuss HIV testing with their partners in the older study [35] compared to the recent one [37]**,** indicating a change in women’s ability to raise such topics and men’s responses.

In regard to the barriers of HTS uptake, fear of testing positive was the one of the most common barrier. Several studies have found that the fear of testing positive for men stems from theirs concerns about losing their masculine characteristics, if diagnosed with HIV, such as being in control of their lives and of their ability to serve as role model and provider for their family [20, 31, 38, 39]. A recent quantitative study conducted with men in the same site found that men who perceived high HIV stigma in their network were less likely to have tested for HIV [26]. Thus, there may be more fear of testing positive in an environment where one may experience stigma, especially for men who perceive themselves to be at high risk for acquiring HIV. Another related fear that has been reported from men in Dar es Salaam and elsewhere is about the lack of privacy and confidentiality at the testing clinic which may lead to their HIV status being shared with community members [20]. In addition to the lack of confidentiality at healthcare facilities, there are also a lack of male friendly services and conflicting hours that are convenient for men who have to work [23].

### Strategies to address HIV testing barriers

Based on the HTS barriers described above and others identified in the Male Catch-Up Plan, non-biomedical and biomedical strategies were developed guide the interventions being implemented to reach men with HTS in Tanzania during the national Test and Treat campaign [23]. The non-biomedical strategies include social and cultural approaches to promote an enabling environment to encourage health seeking behavior (sexual and reproductive health services), safe behavior (sexual risk deduction counseling; comprehensive sexuality education for boys) and providing peer education programs and social marketing to promote condoms. The sexual risk reduction counseling can be offered by peers, at school, or through social media. A recent study found that men who endorse hegemonic masculinity norms, which promote sexual risk-taking among men, are less likely to use condoms consistently in their relationships [40]. In a recently completed RCT in Tanzania, training men as peer educators to engage men in their networks in conversation about reducing inequitable gender norms was found to be effective [41]. Thus, working with men as peer educators to provide counseling and information about equitable gender norms and safe sex is a promising strategy for reducing sexual risk-taking among men, which can alleviate their fear of testing for HIV. Another non-biomedical strategy was to have cultural, political, and religious leaders as champions of male engagement in to HTS. Supporting this strategy, the Prime Minister Kassim Majaliwa tested for HIV in public when he launched the national Test and Treat campaign in June 2018 [42]. The biomedical strategies are described in more details in the following sections.

### Biomedical strategies

The biomedical interventions proposed are aligned with existing government guidelines and programs and based on evidence of what works for men and include: 1) expansion of targeted HIV testing; 2) HIV self-testing; and 3) integration of HTS in other health services.

#### Expansion of targeted HIV testing

Compared to non-targeted screening where all people are offered HIV testing [43], targeted HIV testing is defined as the practice of focusing HTS resources among a particular group that are at high-risk for HIV and unlikely to access routinely-offered HTS [44]. Targeted HTS can be more effective in reaching men than mass HIV testing. The targeted populations are male and female sex workers, men in fishing camps, men who have sex with men, sexual partners, men in construction sites, intravenous drug users, injecting drug users, adolescent boys, long-distance drivers, boda boda operators, and men in uniforms [23]. Targeted HTS can be offered at the health facility or in the community. Research has shown that community HTS achieve higher population coverage than facility testing [45], with home and mobile HTS reaching more men in sub-Saharan Africa [46]. This approach is being used in the ongoing Test and Treat campaign to reach men in different settings [42]. Results from an ongoing President Emergency Plan for AIDS Relief/United States Agency for International Development-funded program called Sauti in Tanzania suggest that targeted HIV testing in hotspots such as bars, brothels, mines and truck stops, or in homes in the case of HIV-exposed partners is an effective approach in increasing HTS [47]. Between October 2016 and September 2017, the Sauti project tested 505,274 and 35,920 of those tested were diagnosed with HIV, suggesting that similar targeted approaches can help community-based HTS efforts reach the first 90 of the 90–90-90 targets among men [47].

#### HIV self-testing

HIVST, which allows a person to perform an HIV test in private or with a trusted person, is another proposed strategy to reach men in Tanzania [23]. HIVST can be conducted at home and delivered in a targeted manner. For example, secondary distribution of HIVST kits from female partners to men in sub-Saharan Africa has been shown to be an acceptable [48], feasible [49], and effective approach for increasing HIV testing among men [50]. Formative research on men’s perceptions on HIVST for the Tanzania STEP (*S*elf-*T*esting *E*ducation and *P*romotion) Project revealed that men are willing to self-test for HIV and reported that HIVST can address some of the barriers to facility-based testing described [28]. Further quantitative analyses showed that having discussed HIV testing with a sexual partner was associated with willingness to self-test for HIV among men who had never been tested for HIV (never-testers), suggesting the potential for sexual partners to deliver HIVST kits to male never-testers while engaging them in HIV testing conversation [30]. In addition, men who had been trained as peer health leaders also expressed their willingness to educate their male peers who did not want to test at the clinic about HIVST [20]. HIVST can help reach the first 90% among adolescent boys as shown in Malawi where 90% of 16–19-years-old male reported to have self-tested during a 2 year community distribution of HIVST trial conducted in 14 neighborhoods (*n* = 16,600) [51]. Based on the effectiveness of HIVST to increase HIV testing [52], the NACP recently started a targeted HIVST demonstration project as part of the Sauti program, with a focus on reaching key populations and male partners of female sex workers through secondary distribution of HIVST [50]. The findings from the demonstration project combined with those from the STEP Project [28] will help inform the national efforts to reach men with HIVST.

#### Integration of HTS into other health services

In addition to expanding targeted HIV testing and provision of HIVST, one other strategy included in the Male Catch-Up Plan is to integrate HTS with other male focused non-communicable diseases (NCDs) such as screening for prostate cancer, voluntary medical male circumcision (VMCC), blood pressure [23]. Integrating HTS with other health services can help reduce the stigma associated with HIV that prevent individuals from testing and leverage the existing infrastructure for HTS to address the increase of NCDs [53, 54]. Other SSA countries have developed similar policies and implemented pilot or nation-wide integration of HTS and NCDs [55]. Based on NIH reporter, a recent study (1R21TW010482–01) funded by the NIH showed that HIV testing nearly doubled in a rural community in Tanzania when an integrated package of screening for HIV, diabetes, and hypertension was offered. Building on these findings, the same research team has an ongoing RCT designed to assess the effect of integrating NCD with HIV screening on HIV testing uptake over 18 months and the effect of adding NCD care to HIV on linkage and enrollment in HIV care as well as retention in care (5R01MH111366–03:PI Sweat). The findings from these studies and those from other countries can help inform the integration of the HIV and NCD screening for men in Tanzania.

Though this study has several strengths since offers a national perspective of the efforts different stakeholders in the country are implementing to address the low uptake of HTS among men, there are limitations regarding the qualitative study and process for developing the Male Catch-Up Plan. First, only a sub-sample of the men participating in the RCT in Dar es Salaam was included in the qualitative study and therefore the findings are not generalizable to all men in Tanzania. Secondly, we relied on men’s self-report of their HIV testing behavior and are not able to confirm men’s HIV testing uptake. For the Male Catch-Up Plan, one of the limitations included the lack of verbatim transcripts since the interviews were not audio recorded and prevented the team from having quotes from the stakeholders for this paper. Another limitation is that despite the team’s effort to conduct a comprehensive analysis of evidence on male engagement and utilization of HIV/AIDS, stakeholders from only two districts were included in the interviews.

## Conclusion

In conclusion, a number of HTS enablers and barriers exist for men in Tanzania. While men’s uptake of HTS has increased over the past decade in Tanzania, men are still not testing at a high enough rate to significantly reduce the number of MLWH who are unaware of their HIV status. In order to reach the UNAIDS 90–90-90 targets a number of strategies have been developed as part of the Male Catch-Up Plan to address some of the HTS barriers and guide the ongoing Test and Treat campaign and other interventions aimed at increasing HTS among men.

## Additional file

Additional file 1: Interview guide. (DOC 42 kb)

## Abbreviations

ART: Antiretroviral Therapy
BMF: Benjamin Mkapa Foundation
CDC: Centers for Disease Control & Prevention
CTC: Care and Treatment Centers
EGPAF: Elizabeth Glaser Pediatric AIDS Foundation
HIV: Human Immunodeficiency Virus
HIVST: HIV Self-Testing
HTS: HIV Testing Services
ICAP: International Centre for AIDS Care and Treatment Program
MLWH: Men Living with HIV
MOHCDGEC: Ministry of Health, Community Development, Gender, Elderly, and Children
NACOPHA: National Council for People Living with HIV and AIDS
NACP: National AIDS Control Program
NCD: Non-Communicable Diseases
NIH: National Institutes of Health
PLWH: People Living with HIV
SSA: Sub-Saharan Africa
STEP: Self-Testing Education & Promotion
TACAIDS: Tanzania Commission for AIDS
UNAIDS: Joint United Nations Program on HIV/AIDS
UNFPA: United Nations Population Fund
VMCC: Voluntary Medical Male Circumcision
WHO: World Health Organization
