# Supplementary material for: Developing national strategies for reaching men with HIV testing services in Tanzania: results from the male catch-up plan
Source: BMC Health Serv Res. 2019 May 20;19:317. doi: 10.1186/s12913-019-4120-3 (PMC6528365; doi:10.1186/s12913-019-4120-3)
Supplement: Supplementary file 1 — Interview guide. (DOC 42 kb) [file 12913_2019_4120_MOESM1_ESM.doc]

INTERVIEW GUIDE

Section 1: Formative research participants

Men who have never tested/NOT tested for HIV in the past 12 months and are willing to self-test

**Introduction:** I would like to thank you for participating in this interview. This interview is part of a study that we are doing with approximately 30 camp members who previously participated in surveys with our Vijana Vijiweni II Project. We have invited you to participate because we would like to ask you more questions to follow-up on topics we asked about in the previous survey you completed with our team. We are interested in learning more about you and your experiences, opinions and beliefs about HIV testing. There are no right or wrong answers in this interview. If there are any questions that make you feel uncomfortable, you should feel free to tell me and we can skip over those questions. We are interested in hearing about your experiences and opinions. Do you have any questions before we begin?

Participant ID_____________

**Socio-demographics**

1. Age____
2. Year of birth____
3. Employment status
4. Self-employed -
5. Employed
6. Unemployed
7. Education
8. No education
9. Primary
10. Secondary
11. Higher than secondary
12. Marital status
13. Single
14. Married
15. Living with partner
16. Camp name _________

**Now, I would like to start asking you a few questions about your HIV testing experiences.**

1. In the previous interview, you mentioned that you had not tested for HIV in the 12 months prior to the interview, **have you ever tested for HIV at any other time previously**?
2. No 🡪 If participant answers No, then **skip questions 8**. Start the Qualitative Interview and

skip questions 3 & 4 in the qualitative interview.

1. Yes 🡪 If participant answers Yes, then ask questions 8 and **skip questions 1 and 2** in the

Qualitative Interview.

1. When was the last time you tested for HIV?

Month______ Year______

**PART I: QUALITATIVE INTERVIEW ON HIV TESTING**

**IF PARTICIPANT HAS NEVER TESTED FOR HIV THEN START HERE AND SKIP QUESTION 3 & 4**

1. Can you please tell me about some of the reason (s) you have not tested for HIV?

Probes:

What goes through your mind when think about not getting tested for HIV?

What goes through your mind when think about getting tested for HIV?

**Thank you for sharing the reasons for not testing for HIV.** Now, I would like to ask about testing for HIV in the future?

1. What, if anything, would make you want you get tested for HIV in the future?

**IF PARTICIPANT HAS TESTED FOR HIV THEN START INTERVIEW HERE.**

Thank you for letting me know you have tested in the past. Now, I would like to ask you some more questions about the last time you tested for HIV.

1. Can you tell me about the reasons you decided to get tested for HIV?

Probes: What made you want to get tested for HIV?

Probes: 1) How did the counselor treat you? 2) Was the counselor a man or woman? 3) How did you feel about the testing experience? Would you go back to the same place for an HIV test in the future?

**Section 2: Barriers and recommendations from stakeholders**

1. What do you think are the barriers/factors affecting uptake of HIV counseling and testing among adolescent boys and adult males in your district?
2. How do you think this problem has to be intervened? Probe on available interventions and if there are other possible interventions at all levels, policy, health sector and community.
